# Multiple-Input-Multiple-Output Filtered Multitone Time Reversal Acoustic Communications Using Direct Adaptation-Based Turbo Equalization

**DOI:** 10.3390/s23136081

**Published:** 2023-07-01

**Authors:** Lin Sun, Haisen Li

**Affiliations:** 1 School of Cyber Science and Engineering, Qufu Normal University, Qufu, 273165, China; 2 College of Underwater Acoustic Engineering, Harbin Engineering University, Harbin, 150001, China

**Keywords:** underwater acoustic communications, filtered multitone, time reversal, adaptive turbo equalization, soft information-based adaptive decision feedback equalization

## Abstract

This paper proposes using direct adaptation (DA)-based turbo equalization in multiple-input-multiple-output (MIMO) filtered multitone (FMT) time reversal (TR) acoustic communications to jointly suppress noise, residual co-channel interference (CCI) and intersymbol interference (ISI) after the TR process. Soft information-based adaptive decision feedback equalization (ADFE) adjusted according to the recursive expected least squares (RELS) algorithm, including interference cancellation and decoding, is used to construct the DA-based turbo equalization. In the proposed method, soft information is exchanged between soft symbols with soft decisions of decoding iteratively, and interference suppression is proceeded successively and iteratively until the performance is stable. The principle of the proposed method is analyzed, and based on the acoustic channel responses measured in a real experiment, the performance is assessed in relation to that of anther two methods. Compared with the MIMO-FMT TR underwater acoustic communication using interference suppression without error control coding (ECC), the proposed method performs better, benefitting from the ECC included in turbo equalization. Additionally, compared with the MIMO-FMT TR underwater acoustic communication using interference suppression based on hard decision equalization and decoding, the proposed method exhibits superior performance by exploiting soft information.

## 1. Introduction

With its high-rate data transmission in marine activities, multiple-input-multiple-output (MIMO) technology has seen widespread use in underwater acoustic communications in the past few decades [1,2,3,4,5,6,7,8,9]. However, MIMO underwater acoustic channels are characterized by long multipath spreads, spatial correlation, serious noise, etc., which can lead to severe co-channel interference (CCI), intersymbol interference (ISI), and noise interference and drastically impact communication performance [10,11].

To achieve high reliability with relatively low-complexity interference suppression, multi-carrier modulation (MCM) has traditionally been exploited in MIMO underwater acoustic communications [1,2,3,4,5] since it can reduce the range of symbols affected by multipath spread and spatial correlation through band splitting. Of all the MCM technologies, orthogonal frequency division multiplexing (OFDM) is the most widely used owing to its simple implementation, and ability to reduce the span of multipath spread and spatial correlation to a symbol interval [1,2,3,4,5]. However, the subbands of OFDM are overlapped and have narrow bandwidth. Additionally, slight frequency offset can lead to severe intercarrier interference (ICI), while complex frequency offset compensation must be adopted in the acoustic communication using OFDM [12,13,14]. In recent years, filtered multitone (FMT), an attractive MCM, has been applied to MIMO underwater acoustic communications [4,5]. Like OFDM, FMT can maintain high spectral efficiency and is easily implemented by using Fast-Fourier transform (FFT). Moreover, in contrast to OFDM, FMT is insensitive to frequency offset, because it has non-overlapping subbands with high spectral containment [15,16]. In MIMO-FMT underwater acoustic communications, offering a compromise between bandwidth efficiency and the complexity of filter implementation, the bandwidth of each subband is still larger than the coherence bandwidth of the acoustic channel. Therefore, although the span of ISI and CCI has been obviously reduced by band splitting, there are still shortened ISI and CCI in demodulated signals of the receiver. Due to its simple implementation and good spatial–temporal compression, time reversal (TR) has been traditionally used as pre-processor to alleviate ISI and CCI in MIMO-FMT acoustic communications [4,5]. However, residual ISI and CCI, as well as noise still exist in the output signals of TR processing; and, therefore, it is necessary to use other technologies as post-processor for TR to further eliminate interference [17]. In [4], a MIMO-FMT TR underwater acoustic communication using successive interference cancellation (SIC) is proposed to remove residual ISI and CCI after TR processing. Although the research has demonstrated that the method presented in [4] can achieve a good communication performance, the suppression of noise has not been considered. 

Deficiencies in existing research motivated us to explore joint suppression of residual ISI and CCI, as well as noise, in MIMO-FMT TR underwater acoustic communications. Generally, applying error control coding (ECC) directly to the method presented in [4] can achieve joint suppression of residual ISI and CCI, as well as noise. However, if interference cancellation, adaptive equalization, and decoding are all based on hard decision, the suppression performance will be limited. Turbo equalization is a powerful interference suppression technique for MIMO underwater acoustic communications [6,7,8,9]. Compared with conventional equalization and decoding based on hard decision, turbo equalization can iteratively exchange soft information of symbols with soft-decision decoding; and, therefore, more information of transmitted bits can be acquired and better communication can be achieved. According to the implementation of adaptive adjustment, turbo equalization can be divided into channel estimation (CE)-based turbo equalization and direct adaptation (DA)-based turbo equalization [18,19]. CE-based turbo equalization can converge quickly, but the computational complexity is relatively high [18]. DA-based turbo equalization needs a longer time to converge, but it can adjust coefficients based on the received signal with low computational complexity; and, therefore, it is more suitable for underwater acoustic communications [20,21,22]. In general, DA-based turbo equalization exploits the decoder soft information as an input to the feedback of adaptive decision feedback equalization (ADFE), rather than using it in adaptive coefficients adjustment. In [23], the DA-based turbo equalization using soft information is presented to improve communication performance, where the soft information is not only exploited in the feedback of ADFE, but also used adaptive coefficients adjustment.

Therefore, based on the advantages of the DA-based turbo equalization using soft information, a MIMO-FMT TR underwater acoustic communication using DA-based turbo equalization is proposed, and the adopted DA-based turbo equalization mainly includes interference cancellation, soft information-based ADFE adjusted according to recursive expected least squares(RELS) algorithm and decoding. In the proposed method, soft information is exchanged between soft symbols with soft decisions of iterative decoding, and the interference suppression proceeds successively and iteratively until the performance is stable. The principle of the proposed method is analyzed, and based on the acoustic channel responses measured in a real experiment, the performance of the proposed method is assessed. Two methods are compared for the performance assessment. The first is the method proposed in [4]. In order to clearly demonstrate the difference from the proposed method, it is referred as the MIMO-FMT TR underwater acoustic communication using interference suppression without error control coding (ECC) in the paper. The second is the MIMO-FMT TR underwater acoustic communication using interference suppression based on hard-decision equalization and decoding, where ECC is introduced to suppress noise, but the equalization and decoding are all based on hard decision without exploiting soft information.

The contribution of the paper is threefold: (1) first, DA-based turbo equalization is first used in MIMO-FMT TR acoustic communications to deal with residual ISI and CCI, as well as noise after TR process; (2) soft information-based ADFE adjusted according to the RELS algorithm along with interference cancellation and decoding is used to construct the adopted DA-based turbo equalization; and (3) based on the acoustic channel responses measured in a real experiment, the performance of the proposed method is compared with that of two other methods to demonstrate its validity. 

This paper is organized as follows. Section 2 describes the system model of the proposed method. Section 3 describes the principle of the DA-based turbo equalization, where the interference cancellation, soft information-based ADFE, and decoding is discussed in detail. Section 4 presents the assessment of the proposed method. Section 5 summarizes the conclusions.

In this work, vectors and matrices are denoted in bold lowercase and uppercase, respectively. Scalars are represented by normal symbols. Conjugate operation, matrix transpose, and Hermitian are denoted by ${\left(\cdot \right)}^{*}$, ${\left(\cdot \right)}^{T}$, and ${\left(\cdot \right)}^{H}$, respectively. $p(x\left|y\right.,z)$ stands for the probability distribution of x conditioned on y, which depends on the value of the deterministic quantity z.

## 2. System Model 

In the paper, for concise analysis, the proposed method is analyzed based on the baseband communication model where two elements simultaneously transmit different user signals and two elements are used to implement diversity reception. It should be noted that based on actual requirements, the proposed method can be applied to communication scenarios with more transmit and receive elements.

### 2.1. Transmitter

**Figure 1:**
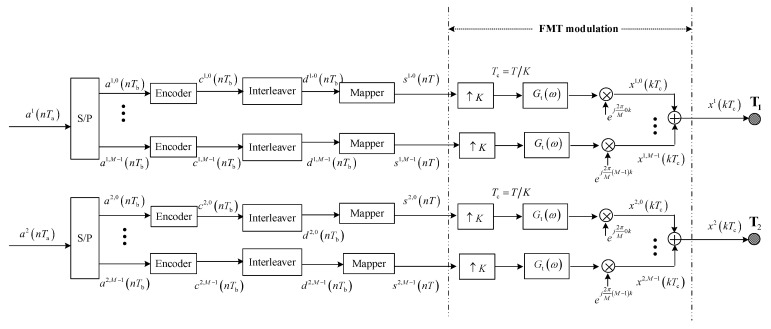
Block diagram for transmit structure.

The transmit structure of the proposed method is depicted in Figure 1. Two information bit streams $\{a^{\alpha }(nT_{a})\},\alpha =1,2$ with the time interval $T_{a}$ are converted into two groups of sub-streams $\{a^{\alpha ,m}(nT_{b})\},\alpha =1,2,m=0,\cdots M-1$ through serial-to-parallel (SP) conversion, where $T_{b}=T_{a}\cdot M$. Then, each bit sub-stream is encoded using convolutional encoders, and the coded bit sub-stream $c^{\alpha ,m}(nT_{b})$ is permuted by interleavers to randomize the order of the code bits. Subsequently, the interleaved bit sub-streams $\{d^{\alpha ,m}(nT_{b})\},\alpha =1,2,m=0,\cdots M-1$ are separately divided into Q-bit blocks and mapped to the symbol sub-sequences from the 2^Q^-ary symbol alphabet S with zero mean and unit energy. Finally, the mapped sub-sequences $\{s^{\alpha ,m}(nT)\},\;\alpha =1,2,m=0,\cdots M-1,T=Q\cdot T_{b}$, are processed by FMT modulation and transmitted from two elements, respectively. 

Referring to FMT modulation in Figure 1, the mapped sub-sequences $\{s^{\alpha ,m}(nT)\},\;\alpha =1,2,m=0,\cdots M-1,T=Q\cdot T_{b}$ are modulated to M subcarriers after *K*-times up-sampling and transmit filtering, and the i-th modulated signal of the α-th group is given by

(1) $$x^{\alpha ,i}(kT_{c})=\sum_{n=-\infty }^{+\infty }s^{\alpha ,i}(nT)g_{t}(kT_{c}-nT)e^{j2\pi f_{i}kT_{c}}$$

where $T_{c}=T/K$ is the time interval after *K*-times up-sampling, $f_{i}$ is the i-th subcarrier, and $g_{t}(kT_{c})$ is the discrete time domain response of the transmit filter. To maintain a balance between implementation complexity and spectral containment, the root raised cosine (RRC)-shaping filter is selected as the transmit filter and the roll-off factor is set to K/M−1.

For total communication bandwidth $B=1/T_{c}$, the intercarrier separation equals $\Delta f=1/T_{c}M$, and then $f_{i}=i\cdot \Delta f=i/T_{c}M$. Substituting $f_{i}$ in Equation (1) with $i/T_{c}M$, $x^{\alpha ,i}(kT_{c})$ can be further expressed as

(2) $$x^{\alpha ,i}(kT_{c})=\sum_{n=-\infty }^{+\infty }s^{\alpha ,i}(nT)g_{t}(kT_{c}-nT)e^{j\frac{2\pi }{M}ik}$$

After combining all modulated signals on M subcarriers, the α-th transmitted signal is given by

(3) $$x^{\alpha }(kT_{c})=\sum_{i=0}^{M-1}\sum_{n=-\infty }^{+\infty }s^{\alpha ,i}(nT)g_{t}(kT_{c}-nT)e^{j\frac{2\pi }{M}ik}$$

### 2.2. Receiver

After transmission through underwater acoustic channels, the signal received by the β-th receive element is

(4) $$y_{\beta }(kT_{c})=\sum_{\rho =-\infty }^{+\infty }\sum_{\alpha =1}^{2}\sum_{i=0}^{M-1}\sum_{n=-\infty }^{+\infty }s^{\alpha ,i}(nT)g_{t}(kT_{c}-\rho T_{c}-nT)h_{\beta }^{\alpha }(\rho T_{c})e^{j\frac{2\pi }{M}i(k-\rho )}+w_{\beta }(kT_{c})$$

where $h_{\beta }^{\alpha }(\rho T_{c})$ denotes the discrete time-domain response of the channel between the α-th transmit element and the β-th receive element, and $w_{\beta }(kT_{c})$ represents channel noise received by the β-th receive element.

**Figure 2:**
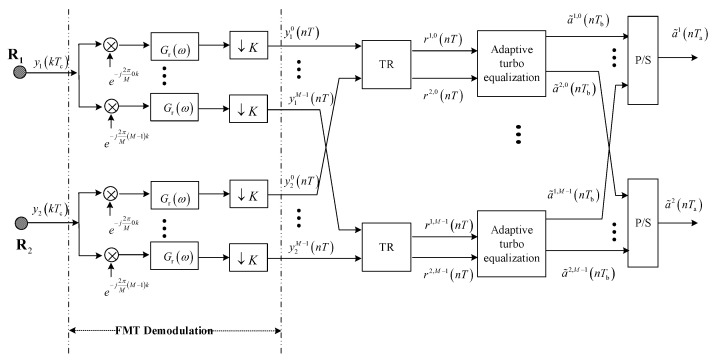
Block diagram for receive structure.

Referring to Figure 2, at the receiver, the received signal $y_{\beta }(kT_{c})$ is processed by FMT demodulation, and the m-th output signal is

(5) $$\begin{array}{l} y_{\beta }^{m}(nT)=\sum_{l=-\infty }^{+\infty }\sum_{\alpha =1}^{2}\sum_{k=-\infty }^{+\infty }\sum_{\rho =-\infty }^{+\infty }g_{t}(kT_{c}-\rho T_{c}-lT)h_{\beta }^{\alpha }(\rho T_{c})e^{-j\frac{2\pi }{M}m\rho }g_{r}(nT-kT_{c})s_{}^{\alpha ,m}(lT)\\ +\sum_{\begin{array}{l} i=0\\ i\neq m \end{array}}^{M-1}\sum_{l=-\infty }^{+\infty }\sum_{\alpha =1}^{2}\sum_{k=-\infty }^{+\infty }\sum_{\rho =-\infty }^{+\infty }g_{t}(kT_{c}-\rho T_{c}-lT)h_{\beta }^{\alpha }(\rho T_{c})e^{-j\frac{2\pi }{M}i\rho }g_{r}(nT-kT_{c})e^{j\frac{2\pi }{M}(i-m)k}s_{}^{\alpha ,i}(lT)\\ +\sum_{k=-\infty }^{+\infty }w_{\beta }(kT_{c})g_{r}(nT-kT_{c})e^{-j\frac{2\pi }{M}mk} \end{array}$$

where $g_{r}(nT_{c})$ refers to the discrete time response of the receive filter.

Observing Equation (5), the second signal component on the right side is the ICI. In FMT, benefitting from non-overlapping subbands with high containment, ICI is a minor concern even with the Doppler spread caused by water motion. This paper’s main concern is joint suppression of residual ISI and CCI, as well as noise in MIMO-FMT TR acoustic communications. The effect of ICI is not taken into consideration. When ICI is neglected, $y_{\beta }^{m}(nT)$ can be simplified as

(6) $$\begin{array}{ll} y_{\beta }^{m}(nT) & =\sum_{l=-\infty }^{+\infty }\sum_{\alpha =1}^{2}\sum_{k=-\infty }^{+\infty }\sum_{\rho =-\infty }^{+\infty }g_{t}(kT_{c}-\rho T_{c}-lT)h_{\beta }^{\alpha }(\rho T_{c})e^{-j\frac{2\pi }{M}m\rho }g_{r}(nT-kT_{c})s_{}^{\alpha ,m}(lT)\\ & +\sum_{k=-\infty }^{+\infty }w_{\beta }(kT_{c})g_{r}(nT-kT_{c})e^{-j\frac{2\pi }{M}mk} \end{array}$$

It can be observed from Equation (6) that the discrete time response of the m-th subchannel between the α-th transmit element and the β-th receive element is

(7) $$h_{\beta }^{\alpha ,m}\left(nT_{c}\right)=h_{\beta }^{\alpha }\left(nT_{c}\right)e^{-j\frac{2\pi }{M}mn}$$

**Figure 3:**
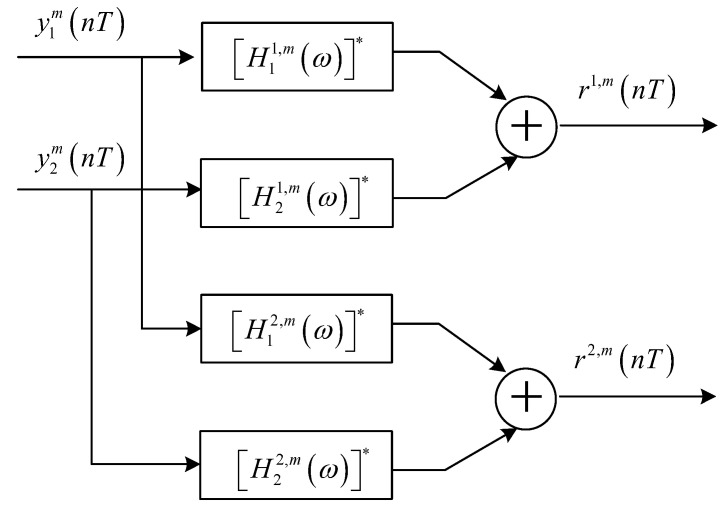
Block diagram for TR processing.

Equation (7) indicates that the responses of subchannels are different and, therefore, the output signals of FMT demodulation should be divided into M groups $\{y_{1}^{m}(nT),y_{2}^{m}(nT)\},m=0,\cdots M-1$ that are processed by TR individually. The procedure of the m-th TR processing is shown in Figure 3, where ${\left[H_{\beta }^{\alpha ,m}(\omega )\right]}^{*}$ denotes the frequency domain response of the filter matched to the frequency domain response of the m-th subchannel between the α-th transmit element and the β-th receive element $H_{\beta }^{\alpha ,m}(\omega )$.

The α-th output signal of the m-th TR processing is

(8) $$r^{\alpha ,m}(nT)=\sum_{l=-\infty }^{+\infty }\sum_{\gamma =1}^{2}q_{}^{\left(\alpha ,\gamma \right),\left(m,m\right)}(lT,nT)s_{}^{\gamma ,m}(lT)+\eta^{\alpha ,m}(nT)$$

where $q_{}^{\left(\alpha ,\gamma ),(m,m\right)}(lT,nT)$ and $\eta^{\alpha ,m}(nT)$ denote the composite channel response and noise after TR processing, and the expressions are as follows:

(9) $$\begin{array}{l} q_{}^{\left(\alpha ,\gamma ),(m,m\right)}(lT,nT)\\ =\sum_{\beta =1}^{2}\sum_{k=-\infty }^{+\infty }\sum_{\varsigma =-\infty }^{+\infty }\sum_{\rho =-\infty }^{+\infty }g_{t}(kT_{c}-\rho T_{c}-lT)h_{\beta }^{\gamma ,m}(\rho T_{c})g_{r}(\varsigma T-kT_{c}){\overset{↼}{h}}_{\beta }^{\alpha ,m}(nT-\varsigma T)\\ \eta^{\alpha ,m}(nT)=\sum_{\beta =1}^{2}\sum_{\varsigma =-\infty }^{+\infty }\sum_{k=-\infty }^{+\infty }w_{\beta }(kT_{c})g_{r}(\varsigma T-kT_{c}){\overset{↼}{h}}_{\beta }^{\alpha ,m}(nT-\varsigma T)e^{-j\frac{2\pi }{M}mk} \end{array}$$

where ${\overset{↼}{h}}_{\beta }^{\alpha ,m}(nT)$ represents the time form corresponding to the frequency domain response ${\left[H_{\beta }^{\alpha ,m}(\omega )\right]}^{*}$.

Equation (8) can be given by

(10) $$\begin{array}{ll} r^{\alpha ,m}(nT) & =q_{}^{\left(\alpha ,\alpha ),(m,m\right)}(nT,nT)s_{}^{\alpha ,m}(nT)+\sum_{l=-\infty }^{+\infty }\sum_{\gamma =1,\gamma \neq \alpha }^{2}q_{}^{\left(\alpha ,\gamma ),(m,m\right)}(lT,nT)s_{}^{\gamma ,m}(lT)\\ & +\sum_{l=-\infty ,l\neq n}^{+\infty }q_{}^{\left(\alpha ,\alpha ),(m,m\right)}(lT,nT)s_{}^{\alpha ,m}(lT)+\eta^{\alpha ,m}(nT) \end{array}$$

Equation (10) shows that in addition to the desired signal component, the output signal of TR processing $r^{\alpha ,m}(nT)$ also contains noise, residual CCI, and ISI that need to be removed.

In the proposed method, DA-based turbo equalization is used as the post-processor, which is described in detail in the following sections. 

## 3. DA-Based Turbo Equalization 

### 3.1. Structure of DA-Based Turbo Equalization

**Figure 4:**
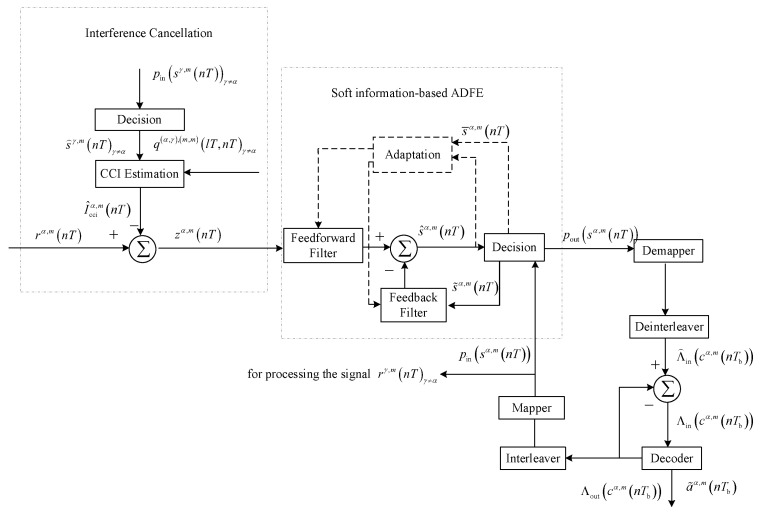
Block diagram for DA-based turbo equalization.

Figure 4 depicts the procedure of DA-based turbo equalization for the signal $r^{\alpha ,m}(nT)$. Referring to Figure 4, interference cancellation and soft information-based ADFE can take advantage of the symbol probabilities $p_{\mathrm{in}}^{}(s^{\alpha ,m}(nT))$ and $p_{\mathrm{in}}^{}(s^{\gamma ,m}(nT)),\;\gamma \neq \alpha$ to undo the effect of residual CCI and ISI. The decoder exploits log likelihood ratios (LLRs) of coded bits $\Lambda_{\mathrm{in}}^{}(c^{\alpha ,m}(nT_{b}))$ to correct error bits caused by noise, the demapper and deinterleaver convert the symbol probabilities $p_{\mathrm{out}}^{}(s^{\alpha ,m}(nT))$ to LLRs of coded bits ${\overset{⌢}{\Lambda }}_{\mathrm{in}}^{}(c^{\alpha ,m}(nT_{b}))$, the mapper and the interleaver convert LLRs of coded bits $\Lambda_{\mathrm{out}}^{}(c^{\alpha ,m}(nT_{b}))$ to the symbol probabilities $p_{\mathrm{in}}^{}(s^{\alpha ,m}(nT))$, and the subtractor is used to ensure that only extrinsic information is passed to the decoder. The soft information exchange and interference suppression are proceeded successively and iteratively until the communication performance is stable. In the following sections, the symbol interval T and the bit interval $T_{b}$ are omitted for concise expression.

### 3.2. Interference Cancellation

In the process of interference cancellation, the residual CCI in the signal $r^{\alpha ,m}(n)$ must first be estimated. Without considering the error of channel estimation, the estimated CCI can be given by

(11) $${\hat{I}}_{cci}^{\alpha ,m}(n)=\sum_{l=-\infty }^{+\infty }\sum_{\gamma =1,\gamma \neq \alpha }^{2}q_{}^{\left(\alpha ,\gamma ),(m,m\right)}(l,n){\overset{⌢}{s}}_{}^{\gamma ,m}(l)$$

where $q_{}^{\left(\alpha ,\gamma ),(m,m\right)}(l,n)$ is the composite channel response given by Equation (9), ${\overset{⌢}{s}}_{}^{\gamma ,m}(n)$ represents the decision value of the symbol $s_{}^{\gamma ,m}(n)$. In the first iteration, ${\overset{⌢}{s}}_{}^{\gamma ,m}(n)$ is set to zero. In the following iteration, ${\overset{⌢}{s}}_{}^{\gamma ,m}(n)$ is computed as ${\overset{⌢}{s}}^{\gamma ,m}(n)=\arg\min_{s\in S}|s-\sum_{s^{\prime }\in S}s^{\prime }p_{\mathrm{in}}(s_{}^{\gamma ,m}(n)=s^{\prime })|$, where s denotes a complex value belonging to the 2^Q^-ary symbol alphabet S and $p_{\mathrm{in}}[s_{}^{\gamma ,m}(n)=s]$ refers to the input a priori probability that $s_{}^{\gamma ,m}(n)=s$.

After removing the estimated CCI from $r^{\alpha ,m}(n)$, the output signal of interference cancellation is

(12) $$\begin{array}{ll} z^{\alpha ,m}(n) & =q_{}^{\left(\alpha ,\alpha ),(m,m\right)}(n,n)s_{}^{\alpha ,m}(n)+\sum_{l=-\infty ,l\neq n}^{+\infty }q_{}^{\left(\alpha ,\alpha ),(m,m\right)}(l,n)s_{}^{\alpha ,m}(l)+\eta^{\alpha ,m}(n)\\ & +\sum_{l=-\infty }^{+\infty }\sum_{\gamma =1,\gamma \neq \alpha }^{2}q_{}^{\left(\alpha ,\gamma ),(m,m\right)}(l,n)s_{}^{\gamma ,m}\left(l\right)-\sum_{l=-\infty }^{+\infty }\sum_{\gamma =1,\gamma \neq \alpha }^{2}q_{}^{\left(\alpha ,\gamma ),(m,m\right)}(l,n){\overset{⌢}{s}}_{}^{\gamma ,m}(l) \end{array}$$

### 3.3. Soft Information-Based ADFE

As shown in Figure 4, the process of soft information-based ADFE consists of signal filtering represented by solid lines and adaptive coefficient adjustment indicated by dashed lines.

#### 3.3.1. Signal Filtering

Specifically, define

(13) $$\begin{array}{l} z^{\alpha ,m}(n)={\left[z^{\alpha ,m}(n),\ldots ,z^{\alpha ,m}(n-(N_{f}-1))\right]}^{T}\\ {\tilde{s}}_{}^{\alpha ,m}(n)={\left[{\tilde{s}}^{\alpha ,m}(n),\ldots ,{\tilde{s}}^{\alpha ,m}(n-(N_{b}-1))\right]}^{T} \end{array}$$

where $N_{f}$ and $N_{b}$ refer to the length of the feedforward filter and feedback filter, respectively; and ${\tilde{s}}^{\alpha ,m}(n)$ denotes the decision fed into the feedback filter. In the first iteration, the value of ${\tilde{s}}_{}^{\alpha ,m}(n)$ is equal to the hard decision of filtered output signal ${\hat{s}}_{}^{\alpha ,m}(n)$, and in the subsequent iteration, ${\tilde{s}}_{}^{\alpha ,m}(n)=\sum_{s\in S}sp_{\mathrm{in}}(s_{}^{\alpha ,m}(n)=s)$. 

The input vector of signal filtering is defined as

(14) $$u^{\alpha ,m}(n)=\left[\begin{array}{l} z^{\alpha ,m}(n)\\ {\tilde{s}}_{}^{\alpha ,m}(n) \end{array}\right]$$

The filtered output signal can be written as

(15) $${\hat{s}}_{}^{\alpha ,m}(n)={\left[f^{\alpha ,m}(n)\right]}^{H}u^{\alpha ,m}(n)={\left[\begin{array}{l} f_{f}^{\alpha ,m}(n)\\ -f_{b}^{\alpha ,m}(n) \end{array}\right]}^{H}\left[\begin{array}{l} z^{\alpha ,m}(n)\\ {\tilde{s}}_{}^{\alpha ,m}(n) \end{array}\right]$$

where $f^{\alpha ,m}(n)$ is the filter coefficient vector, including feedforward-filter coefficients $f_{f}^{\alpha ,m}(n)$ and feedback-filter coefficients $f_{b}^{\alpha ,m}(n)$.

#### 3.3.2. Adaptive Coefficient Adjustment

In the proposed method, the RELS algorithm [23] is adopted for adaptive coefficient adjustment. The update steps are as follows. 

(1) Computing the Kalman gain,

(16) $$k_{}^{\alpha ,m}(n)=\frac{J_{}^{\alpha ,m}(n-1)u^{\alpha ,m}(n)}{\lambda +{\left[u^{\alpha ,m}(n)\right]}^{H}J_{}^{\alpha ,m}(n-1)u^{\alpha ,m}(n)}$$

where $J_{}^{\alpha ,m}(n-1)$ denotes the inverse covariance matrix at time n−1, λ is the forgetting factor.

(2) Updating the coefficient vector

(17) $$f^{\alpha ,m}(n+1)=f^{\alpha ,m}(n)+k_{}^{\alpha ,m}(n){\left[{\bar{s}}_{}^{\alpha ,m}(n)-{\hat{s}}_{}^{\alpha ,m}(n)\right]}^{*}$$

where ${\bar{s}}_{}^{\alpha ,m}(n)$ is the decision value used for adaptive coefficient adjustment. When ADFE operates in training mode, ${\bar{s}}_{}^{\alpha ,m}(n)$ is equal to $s_{}^{\alpha ,m}(n)$, and when ADFE operates in decision directed mode, ${\bar{s}}_{}^{\alpha ,m}(n)$ is computed as

(18) $${\bar{s}}_{}^{\alpha ,m}(n)=\frac{\sum_{s\in S}sp[{\hat{s}}_{}^{\alpha ,m}(n)\left|{\hat{s}}_{}^{\alpha ,m}(n-1),s_{}^{\alpha ,m}(n)=s;f^{\alpha ,m}(n)\right.]p_{\mathrm{in}}[s_{}^{\alpha ,m}(n)=s]}{\sum_{s\in S}p[{\hat{s}}_{}^{\alpha ,m}(n)\left|{\hat{s}}_{}^{\alpha ,m}(n-1),s_{}^{\alpha ,m}(n)=s;f^{\alpha ,m}(n)\right.]p_{\mathrm{in}}[s_{}^{\alpha ,m}(n)=s]}$$

where ${\hat{s}}_{}^{\alpha ,m}(n-1)=\{{\hat{s}}_{}^{\alpha ,m}(n-1),\ldots ,{\hat{s}}_{}^{\alpha ,m}(1)\}$, and in the first iteration, the symbols are always assumed to be equal probabilities, i.e., $p_{in}[s_{}^{\alpha ,m}(n)=s]=1/2^{Q}$.

(3) Updating the inverse covariance matrix

(19) $$J_{}^{\alpha ,m}(n)=\frac{J_{}^{\alpha ,m}(n-1)-k_{}^{\alpha ,m}(n){\left[u^{\alpha ,m}(n)\right]}^{H}J_{}^{\alpha ,m}(n-1)}{\lambda }$$

Based on observations made in relation to Equation (18), for computing ${\bar{s}}_{}^{\alpha ,m}(n)$, a suitable model for the distribution $p[{\hat{s}}_{}^{\alpha ,m}(n)\left|{\hat{s}}_{}^{\alpha ,m}(n-1),s_{}^{\alpha ,m}(n)=s;f^{\alpha ,m}(n)\right.]$ must be selected. The research presented in [24] shows that ignoring the information about $s_{}^{\alpha ,m}(n)$ provided by ${\hat{s}}_{}^{\alpha ,m}(n-1),\ldots {\hat{s}}_{}^{\alpha ,m}(1)$ only adds some noise following nearly Gaussian distribution; and, therefore, $p[{\hat{s}}_{}^{\alpha ,m}(n)\left|{\hat{s}}_{}^{\alpha ,m}(n-1),s_{}^{\alpha ,m}(n)=s;f^{\alpha ,m}(n)\right.]$ can be approximated by $p[{\hat{s}}_{}^{\alpha ,m}(n)\left|s_{}^{\alpha ,m}(n)=s;f^{\alpha ,m}(n)\right.]$. 

Substituting $p[{\hat{s}}_{}^{\alpha ,m}(n)\left|{\hat{s}}_{}^{\alpha ,m}(n-1),s_{}^{\alpha ,m}(n)=s;f^{\alpha ,m}(n)\right.]$ by $p[{\hat{s}}_{}^{\alpha ,m}(n)\left|s_{}^{\alpha ,m}(n)=s;f^{\alpha ,m}(n)\right.]$, ${\bar{s}}_{}^{\alpha ,m}(n)$ can be computed as

(20) $${\bar{s}}_{}^{\alpha ,m}(n)=\frac{\sum_{s\in S}sp[{\hat{s}}_{}^{\alpha ,m}(n)\left|s_{}^{\alpha ,m}(n)=s;f^{\alpha ,m}(n)\right.]p_{\mathrm{in}}[s_{}^{\alpha ,m}(n)=s]}{\sum_{s\in S}p[{\hat{s}}_{}^{\alpha ,m}(n)\left|s_{}^{\alpha ,m}(n)=s;f^{\alpha ,m}(n)\right.]p_{\mathrm{in}}[s_{}^{\alpha ,m}(n)=s]}$$

where $p[{\hat{s}}_{}^{\alpha ,m}(n)\left|s_{}^{\alpha ,m}(n)=s;f^{\alpha ,m}(n)\right.]$ is assumed to be a Gaussian distribution with mean s and variance $\sigma_{}^{2}|{\hat{s}}^{\alpha ,m}(n)$ estimated using a variance estimator presented in [24].

### 3.4. Decoding

After processing by decision device, the signal ${\hat{s}}_{}^{\alpha ,m}(n)$ is mapped into the output probabilities on the possible symbols. For each s belonging to the symbol alphabet S, the output probabilities can be computed as

(21) $$p_{\mathrm{out}}(s_{}^{\alpha ,m}(n)=s)=\frac{p[{\hat{s}}_{}^{\alpha ,m}(n)\left|s_{}^{\alpha ,m}(n)=s;f^{\alpha ,m}(n)\right.]p_{\mathrm{in}}[s_{}^{\alpha ,m}(n)=s]}{\sum_{s\in S}p[{\hat{s}}_{}^{\alpha ,m}(n)\left|s_{}^{\alpha ,m}(n)=s;f^{\alpha ,m}(n)\right.]p_{\mathrm{in}}[s_{}^{\alpha ,m}(n)=s]}$$

The output probabilities are sequentially processed by the soft demapper and deinterleaver, and LLRs of coded bits for the decoder input can be obtained,

(22) $${\overset{⌢}{\Lambda }}_{\mathrm{in}}^{}(c^{\alpha ,m}(n))=\log\frac{p_{\mathrm{in}}(c^{\alpha ,m}(n)=1)}{p_{\mathrm{in}}(c^{\alpha ,m}(n)=0)}$$

To ensure that only extrinsic information is transferred, LLRs of coded bits ${\overset{⌢}{\Lambda }}_{\mathrm{in}}^{}(c^{\alpha ,m}(n))$ are passed to the decoder after subtracting the last output LLRs of the decoder $\Lambda_{\mathrm{out}}^{}(c^{\alpha ,m}(n))$. In the first iteration, the last output LLRs of the decoder is set to 0. 

## 4. Performance Assessment

### 4.1. Channel Responses Measured in an Actual Experiment

The performance of the proposed method was assessed based on a group of channel responses measured in an MIMO-FMT TR acoustic communication experiment carried out in an indoor pool of 45 m in length, 6 m in width, and 5 m in depth. Four sides of the pool were covered with acoustic absorbent materials. The surface and bottom reflected the acoustic signal. In the experiment, two hemispherical transducers placed at 1.5 m and 2 m below the surface were used to transmit two user signals. Two spherical hydrophones deployed at 0.7 m and 0.9 m below the surface provided a vertical array for receiving. The communication distance was 15 m. The other parameters settings are shown in Table 1. 

In the experiment, the input signal-to-noise ratio (SNR) at the receiver was approximately 24 dB and, therefore, the effect of noise on channel estimation was very mild and could be ignored. 

**Figure 5:**
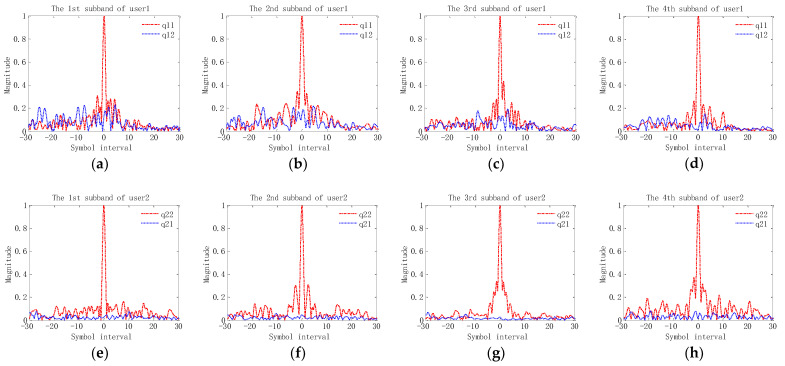
Composite channel responses after TR process: (**a**–**d**) the channel responses of user1 from the 1st subband to the 4th subband; (**e**–**h**) the channel responses of user2 from the 1st subband to the 4th subband.

Figure 5 shows the measured composite channel responses for two users after the TR process. In Figure 5, $q_{}^{\left(\alpha ,\alpha ),(m,m\right)}(t)$ represented by red dash-dotted line is a continuous time form corresponding to the composite channel response $q_{}^{\left(\alpha ,\alpha ),(m,m\right)}(lT,nT)$, which is normalized with respect to its maximum amplitude; $q_{}^{\left(\alpha ,\gamma ),(m,m\right)}(t),\alpha \neq \gamma$ represented by the blue dotted line, is the continuous time form corresponding to the composite channel response $q_{}^{\left(\alpha ,\gamma ),(m,m\right)}(lT,nT)$ and that is normalized with respect to the maximum amplitude of $q_{}^{\left(\alpha ,\alpha ),(m,m\right)}(t)$. Combined with Equation (9), the amplitude of the mainlobe in $q_{}^{\left(\alpha ,\alpha ),(m,m\right)}(t)$ reflects the intensity of the desired signal, the range of sidelobes in $q_{}^{\left(\alpha ,\alpha ),(m,m\right)}(t)$ indicates the intensity and range of ISI, and the amplitude and range of $q_{}^{\left(\alpha ,\gamma ),(m,m\right)}(t),\alpha \neq \gamma$ shows the intensity and range of CCI. Two observations can be obtained from Figure 5. Firstly, although the side lobe of $q_{}^{\left(\alpha ,\alpha ),(m,m\right)}(t)$ is much smaller than its main lobe, it is not fully compressed, and the symbol range of impact is relatively large. This observation indicates that the residual ISI after TR processing is still relatively large. Secondly, the amplitude of $q_{}^{\left(\alpha ,\gamma ),(m,m\right)}(t),\alpha \neq \gamma$ is much lower than the main lobe of $q_{}^{\left(\alpha ,\alpha ),(m,m\right)}(t)$, but it has not been compressed completely. This observation reveals that there is still residual CCI that needs to be removed. 

### 4.2. Two Methods for Comparison 

In order to assess performance, two methods for comparison are also analyzed. In the following sections, for simplicity of expression, two methods for comparison are abbreviated as the method without ECC and the method based on hard decision, respectively.

**Figure 6:**
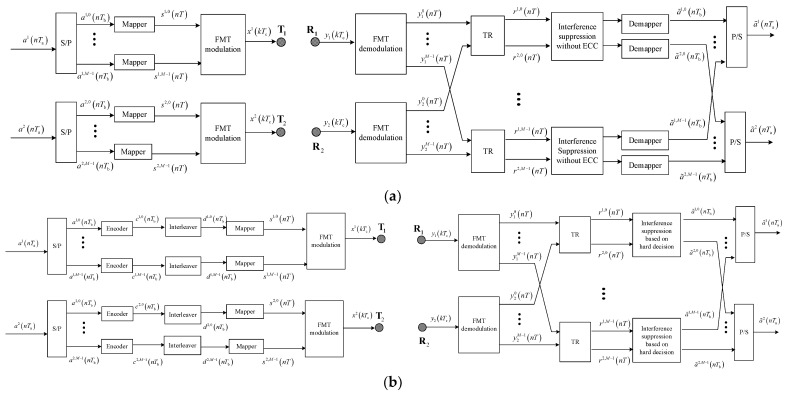
Block diagram for communication structure of two methods for comparison: (**a**) the method without ECC; (**b**) the method based on hard decision.

**Figure 7:**
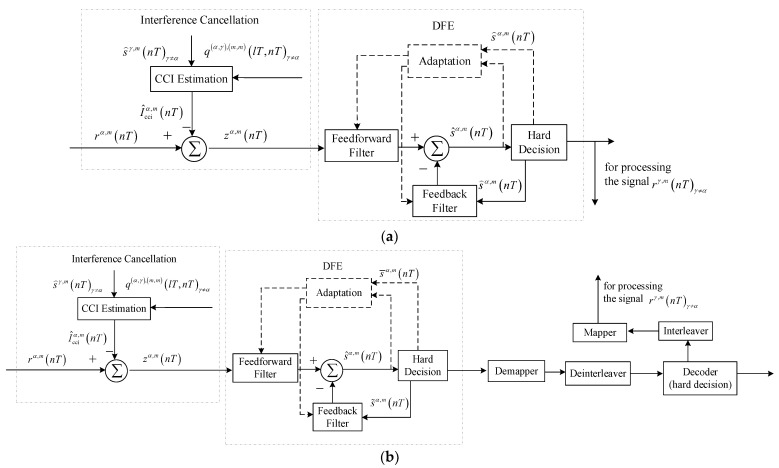
Block diagram for interference suppression of two methods for comparison: (**a**) the method without ECC; (**b**) the method based on hard decision.

The communication structure and interference suppression of two methods for comparison are shown in Figure 6 and Figure 7; the structure of FMT modulation and demodulation are shown in Figure 1 and Figure 2. 

### 4.3. Parameters Setting

Based on the channel responses measured in the real experiment mentioned above, the proposed method is compared with the method without ECC and the method based on hard decision through simulation analysis. In the simulation, the communication frequency band, the carrier modulation mode, the number of subbands, the roll-off factor of transmit filter, and the symbol rate on each subband were the same as those for the real experiment shown in Table 1. The other parameters are shown in Table 2. 

Referring to Table 2, the rate R=1/2 recursive systemic code (RSC) with generator polynomial [5,7] is used for coding; and, therefore, in order to compare three methods with the same information rate, quadrature phase shift keying (QPSK) is used in the proposed method and the method based on hard decision, while BPSK is adopted in the method without ECC.

### 4.4. Comparison between the Proposed Method and the Method without ECC 

**Figure 8:**
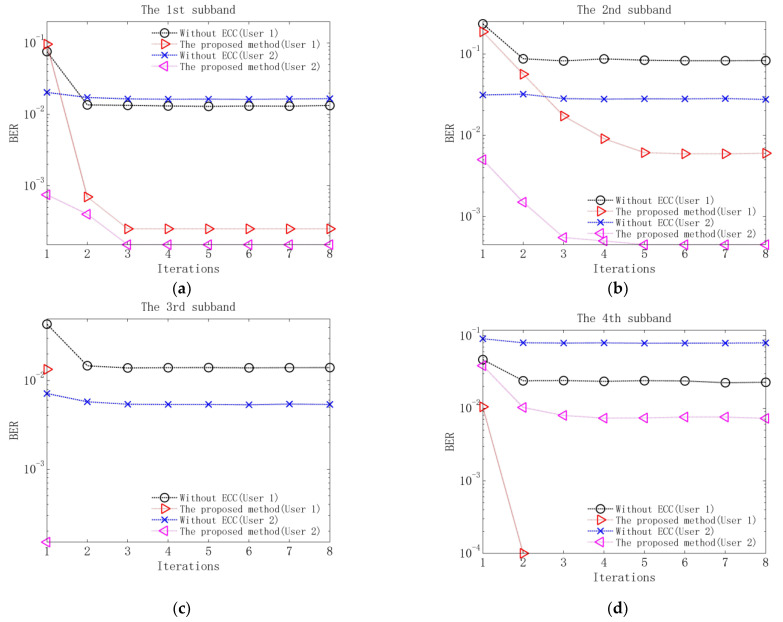
BERs of the proposed method and the method without ECC at input SNR of 10 dB: (**a**) BERs of the 1st subband; (**b**) BERs of the 2nd subband; (**c**) BERs of the 3rd subband; (**d**) BERs of the 4th subband.

Performance comparison is measured using the output bit-error-rate (BER) of each subband decoder of the proposed method and the output BER of each subband equalizer of the method without ECC. The simulation results without additional noise are shown in Table 3, and the results at input SNR of 10 dB acquired by adding white Gaussian noise to the signal passing through each subband are shown in Figure 8. The noise variance $\sigma_{m}^{2}$ is determined according to the $SNR=\sum {\left|h_{\beta }^{\alpha ,m}(n)\right|}^{2}/2\sigma_{m}^{2}\mathrm{QR}$. 

It can be observed from Table 3 that after three iterations, the BERs of two users in the proposed method all reach zero, but the BERs of the 2nd subband of two users and the 4th subband of user 2 in the method without ECC still remain at the order of magnitudes $10^{-4}$. The observations indicate when the residual ISI and CCI are the main factors degrading communication performance and noise is ignored, the proposed method can obtain better performance than the method without ECC due to its superior residual ISI and CCI suppression. Figure 8 shows that at input SNR of 10 dB, when the performance of two methods reaches stability, the BERs of the proposed method are obviously lower than those of the method without ECC. This implies that when noise, residual ISI and CCI are the main factors injuring communication performance, the advantage of the proposed method shows a more pronounced benefit from the use of ECC. 

### 4.5. Comparison between the Proposed Method and the Method Based on Hard Decision 

**Figure 9:**
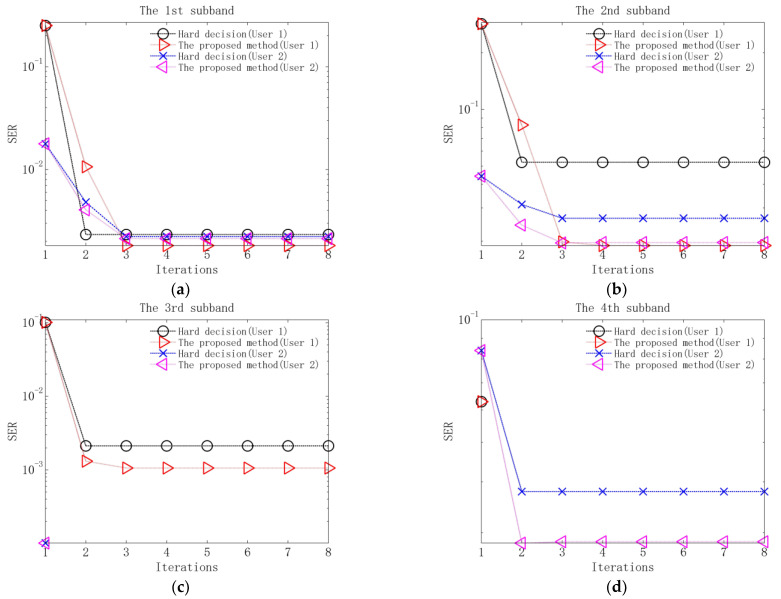
Output SERs of equalization of the proposed method and the method based on hard decision in the case of no added noise: (**a**) SERs of the 1st subband; (**b**) SERs of the 2nd subband; (**c**) SERs of the 3rd subband; (**d**) SERs of the 4th subband.

**Figure 10:**
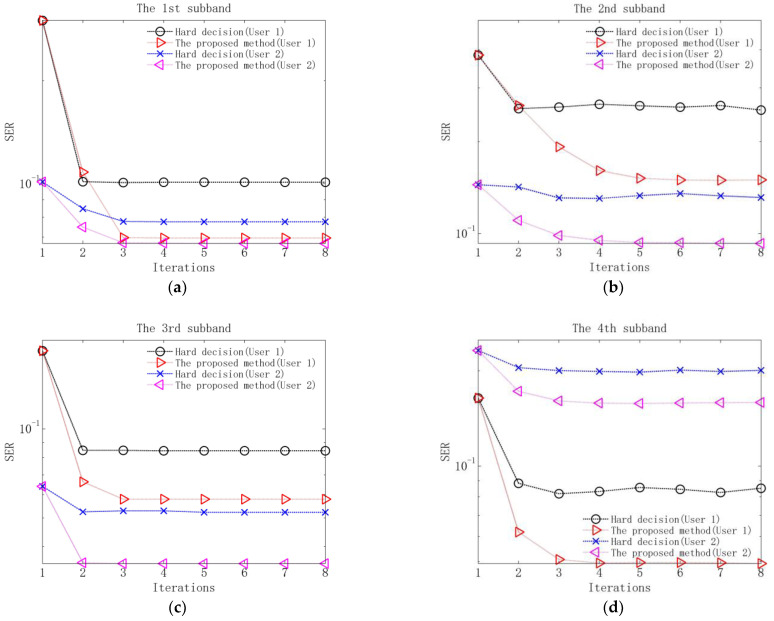
Output SERs of equalization of the proposed method and the method based on hard decision at input SNR of 10 dB: (**a**) SERs of the 1st subband; (**b**) SERs of the 2nd subband; (**c**) SERs of the 3rd subband; (**d**) SERs of the 4th subband.

**Figure 11:**
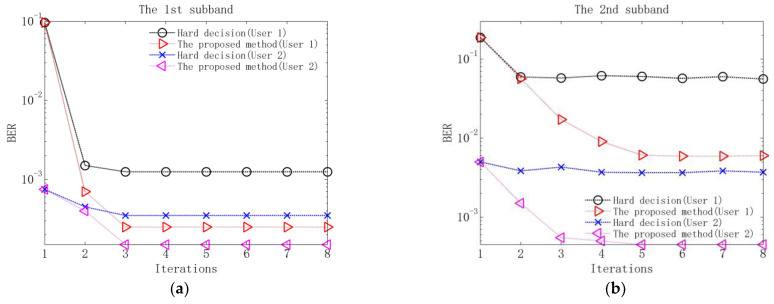

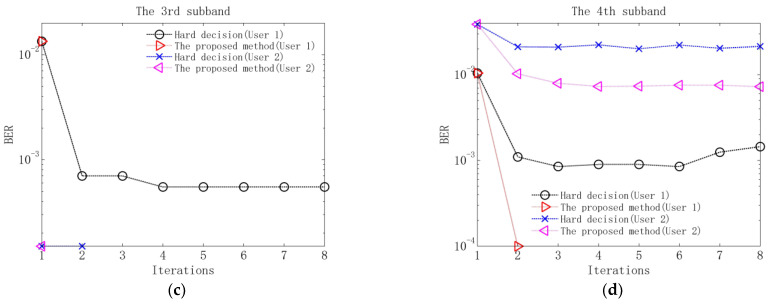
Output BERs of decoding of the proposed method and the method based on hard decision at 10 dB input SNR: (**a**) BERs of the 1st subband; (**b**) BERs of the 2nd subband; (**c**) BERs of the 3rd subband; (**d**) BERs of the 4th subband.

Performance comparison between the proposed method and the method based on hard decision is measured using the output symbol error rate (SER) of each equalizer and the output BER of each decoder. The results without additional noise are shown in Figure 9 and Table 4, and the results when input SNR is 10 dB are shown in Figure 10 and Figure 11.

Several observations can be obtained from Figure 9, Figure 10 and Figure 11 and Table 4. Firstly, in the first iteration, the two methods have the same performance after equalization and decoding. The reason is that in the first iteration, due to the lack of soft information, the equalization and decoding of the proposed method are based on hard decision. Secondly, when the equalization performance of the two methods tends to be stable after 2–3 iterations, the output SERs of the proposed method are lower than those of the method based on hard decision. The reason is that the proposed method utilizes soft information in the process of interference cancellation and equalization, so it has better residual ISI and CCI suppression performance. Thirdly, when the decoding performance of the two methods is stable, the output BERs of the proposed method are lower than those of the method based on hard decision. The reason is that the proposed method exploits the soft information in the decoding, so better error correction performance can be obtained.

## 5. Conclusions

In this paper, the MIMO-FMT TR acoustic communication using DA-based turbo equalization has been proposed to jointly suppress noise, residual CCI and ISI after the TR process. In the proposed method, the adopted DA-based turbo equalization is constructed via interference cancellation and soft information-based ADFE adjusted according to the RELS algorithm and decoding. Soft information exchange and interference suppression are proceeded successively and iteratively until the communication performance is stable. The principle of the proposed method is analyzed, and based on the acoustic channel responses measured in a real experiment, the performance of the proposed method is compared with that of two methods. The results show that compared with the MIMO-FMT TR underwater acoustic communication using interference suppression without error control coding (ECC), the proposed method performs better, benefitting from the ECC included in turbo equalization; and compared with the MIMO-FMT TR underwater acoustic communication using interference suppression based on hard-decision equalization and decoding, the proposed method can achieve superior performance due to the exploitation of soft information.

## Figures and Tables

**Table 1:** Parameters of the experiment.

Parameters	Setting
Communication frequency band	8–16 kHz
Carrier modulation mode	FMT
The number of subbands	4
Roll-off factor	0.5
Symbol rate on each subband	4000/3
Constellation	Binary phase shift keying (BPSK)
Number of symbol for channel estimation	250
Algorithm for channel estimation	Recursive least square
Forgetting factor	0.999

**Table 2:** Parameters of the proposed method and two methods for comparison.

Parameters	The Proposed Method	The Method without ECC	The Method Based on Hard Decision
Number of information bits for each user	20,000	20,000	20,000
ECC	The rate 1/2 RSC with generator polynomial [5,7]	none	The rate 1/2 RSC with generator polynomial [5,7]
Constellation	QPSK	BPSK	QPSK
Number of mapped symbols for each user	20,000	20,000	20,000
Total information rate of each user	16,000/3	16,000/3	16,000/3
Feedforward filter taps of each DFE	23	23	23
Feedback filter taps of each DFE	8	8	8
Adaptive equalization algorithm	RLSE	RLS	RLS
Decoding algorithm	Log-MAP	None	Viterbi

**Table 3:** BERs for the proposed method and the method without ECC in the case of no added noise.

Method	Subband Index	The 1st Iteration (User1/User2)	The 2nd Iteration (User1/User2)	The 3rd Iteration (User1/User2)	The 4th–8th Iterations (User1/User2)
The method without ECC	1	2.0 × 10^−2^/7.6 × 10^−5^	0/0	0/0	0/0
	2	7.8 × 10^−2^/1.3 × 10^−3^	2.2 × 10^−4^/6.1 × 10^−4^	1.5 × 10^−4^/4 × 10^−4^	1.3 × 10^−4^/4 × 10^−4^
	3	4.1 × 10^−3^/0	0/0	0/0	0/0
	4	1.8 × 10^−3^/4 × 10^−3^	0/6.8 × 10^−4^	0/6.5 × 10^−4^	0/6.5 × 10^−4^
The proposed method	1	4.4 × 10^−2^/0	0/0	0/0	0/0
	2	7.4 × 10^−2^/0	5 × 10^−4^/0	0/0	0/0
	3	3 × 10^−4^/0	0/0	0/0	0/0
	4	0/0	0/0	0/0	0/0

**Table 4:** Output BERs of decoding of the proposed method and the method based on hard decision in the case of no added noise.

Method	Subband Index	The 1st Iteration (User1/User2)	The 2nd Iteration (User1/User2)	The 3rd Iteration (User1/User2)	The 4th–8th Iterations (User1/User2)
The method based on hard decision	1	4.4 × 10^−2^/0	0/0	0/0	0/0
	2	7.4 × 10^−2^/0	1.5 × 10^−4^/0	1.5 × 10^−4^/0	1.5 × 10^−4^/0
	3	3.0 × 10^−4^/0	0/0	0/0	0/0
	4	0/0	0/0	0/0	0/0
The proposed method	1	4.4 × 10^−2^/0	0/0	0/0	0/0
	2	7.4 × 10^−2^/0	5 × 10^−4^/0	0/0	0/0
	3	3.0 × 10^−4^/0	0/0	0/0	0/0
	4	0/0	0/0	0/0	0/0

## Data Availability

The data presented in this paper are available after contacting the corresponding author.
